# In Love with Shaping You—Influential Factors on the Breast Milk Content of Human Milk Oligosaccharides and Their Decisive Roles for Neonatal Development

**DOI:** 10.3390/nu12113568

**Published:** 2020-11-20

**Authors:** Christian Hundshammer, Oliver Minge

**Affiliations:** Wacker Chemie AG, Biosolutions, Hanns-Seidel-Platz 4, 81737 München, Germany; oliver.minge@wacker.com

**Keywords:** human milk oligosaccharides, milk sugars, breast feeding, infant food, prebiotics, antiadhesive, intestinal barrier, immune system, milk content

## Abstract

Human milk oligosaccharides (HMOs) are structurally versatile sugar molecules constituting the third major group of soluble components in human breast milk. Based on the disaccharide lactose, the mammary glands of future and lactating mothers produce a few hundreds of different HMOs implicating that their overall anabolism utilizes rather high amounts of energy. At first sight, it therefore seems contradictory that these sugars are indigestible for infants raising the question of why such an energy-intensive molecular class evolved. However, in-depth analysis of their molecular modes of action reveals that Mother Nature created HMOs for neonatal development, protection and promotion of health. This is not solely facilitated by HMOs in their indigestible form but also by catabolites that are generated by microbial metabolism in the neonatal gut additionally qualifying HMOs as natural prebiotics. This narrative review elucidates factors influencing the HMO composition as well as physiological roles of HMOs on their way through the infant body and within the gut, where a major portion of HMOs faces microbial catabolism. Concurrently, this work summarizes in vitro, preclinical and observational as well as interventional clinical studies that analyzed potential health effects that have been demonstrated by or were related to either human milk-derived or synthetic HMOs or HMO fractions.

## 1. Structure of Human Milk Oligosaccharides and Human Milk Content

**Figure 1:**
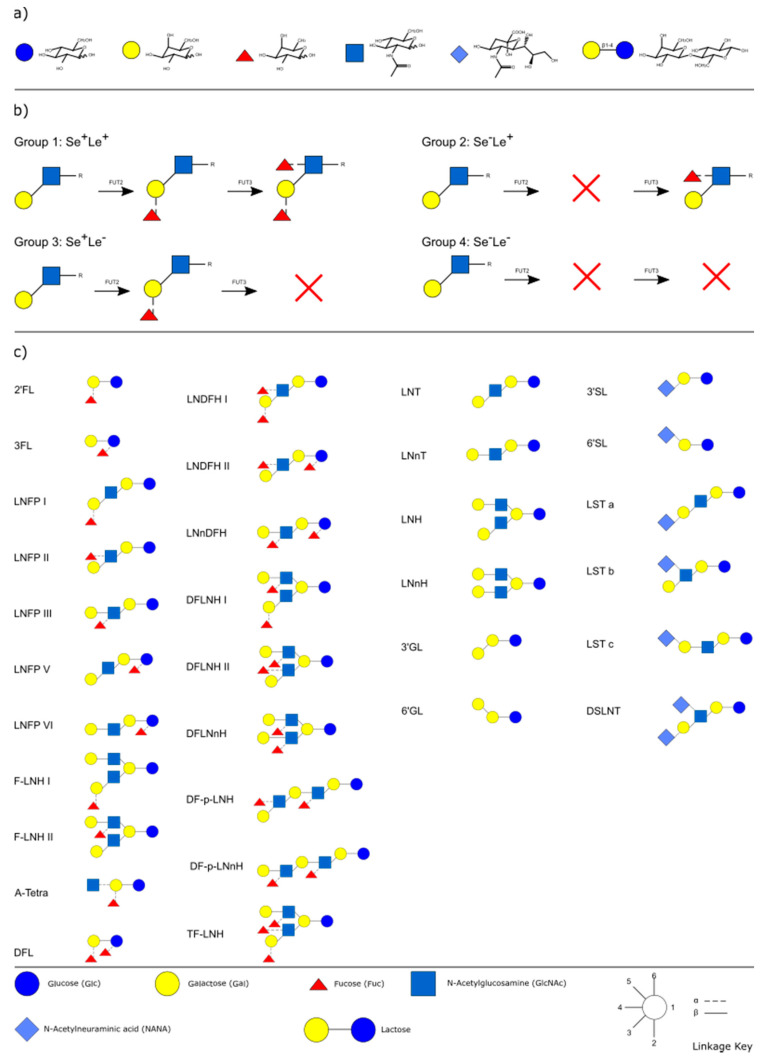
(**a**) Basic building blocks of human milk oligosaccharides; (**b**) effect of Secretor and Lewis genes on the composition of HMOs; and (**c**) main neutral fucosylated, neutral non-fucosylated and acidic HMOs. Abbreviations: 2′FL: 2′-fucosyllactose; 3FL: 3-fucosyllactose; LNFP I, II, III, IV, V, VI: lacto-N-fucopentaose I, II, II, IV, V, IV; F-LNH I, II: fucosyl-lacto-N-hexaose I, II; A-Tetra: A-tetrasaccharide; DFL: difucosyllactose; LNDFH I, II: lacto-N-difucosylhexaose I, II; LNnDFH: lacto-N-neodifucosylhexaose; DFLNH I, II: difucosyllacto-N-hexaose I, II; DFLNnH: difucosyllacto-N-neohexaose; DF-p-LNH: difucosyl-para-lacto-N-hexaose; DF-p-LNnH: difucosyl-para-lacto-N-neohexaose; TF-LNH: trifucosyl-lacto-N-hexaose; LNT: lacto-N-tetraose; LNnT: lacto-N-neotetraose; LNH: lacto-N-hexaose; LNnH: lacto-N-neohexaose; 3′/6′GL: 3′-/6′-galactosyloligosaccharide; 3′/6′SL: 3′-/6′- sialyllactose; LST a, b, c: sialyllacto-N-tetraose a, b, c; DSLNT: disialyllacto-N-tetraose.

Human milk oligosaccharides (HMOs) are lactose-derived molecules constituting a major part of human breast milk. Even though they are highly varied in structure, the oligosaccharides are composed of only five building blocks namely glucose (Glc), galactose (Gal), fucose (Fuc), N-acetylglucosamine (GlcNAc) and N-acetylneuraminic acid (NANA, Figure 1a).

Depending on their fucose and L-N-acetylneuraminic acid (=sialic acid) content, HMOs can be further divided into the three groups of neutral fucosylated, neutral non-fucosylated and acidic sialylated HMOs. Based on studies that performed absolute quantification, these respectively make up 35–77%, 11–30% and 4–27% [1,2,3,4,5] of total HMOs and overall form nearly 200 different molecules [2]. Note that some studies state that more than 200 HMOs have already been identified. However, to the best of our knowledge, this has not been shown so far.

The mean total HMO content in human milk ranges from 4 g/L [6] to 22 g/L (Table 1), while individual mothers might produce even greater amounts of up to more than 30 g/L [5,7]. This high variability is due to several intrinsic and extrinsic factors that differ drastically between individual mother/infant pairs.

One of the most important intrinsic factors is the genetic predisposition of so-called glycosyltransferases, namely α-1,2-fucosyltransferase (FUT2) and α-1,3/4-fucosyltransferase (FUT3). These enzymes link fucose to terminal galactose residues or subterminal GlcNAc residues while different expression activities of the underlying Secretor (Se) and Lewis (Le) genes generally result in the four phenotypes frequently called milk groups: Se^+^Le^+^, Se^+^Le^−^, Se^−^Le^+^ and Se^−^Le^−^ (Figure 1b).

Studies that differentiated mothers according to their milk group by means of breastmilk analysis are summarized in Table 2. Secretor- and Lewis-positive (Se^+^Le^+^) mothers have been shown to be the most dominant group (45–77%) in several populations from Europe, Asia and Africa. The second most dominant group is the non-Secretor Lewis-positive group (Se^−^Le^+^, 7–34%), followed by the Secretor Lewis-negative group (Se^+^Le^−^, 4–28%) and the non-Secretor Lewis-negative group (Se^−^Le^−^, 1–26%) [10].

In addition to this grouping, fucosyltransferases other than FUT2/3 seem to influence the HMO composition, as milk of Secretor- and Lewis-deficient mothers also contains small amounts of fucosylated HMOs [7,11,12]. This is in agreement with van Leeuwen et al., who have recently suggested that the fucosyltransferase FucTx is responsible for regional variations of the HMO composition of Lewis-negative mothers in Vietnam [13].

**Figure 2:**
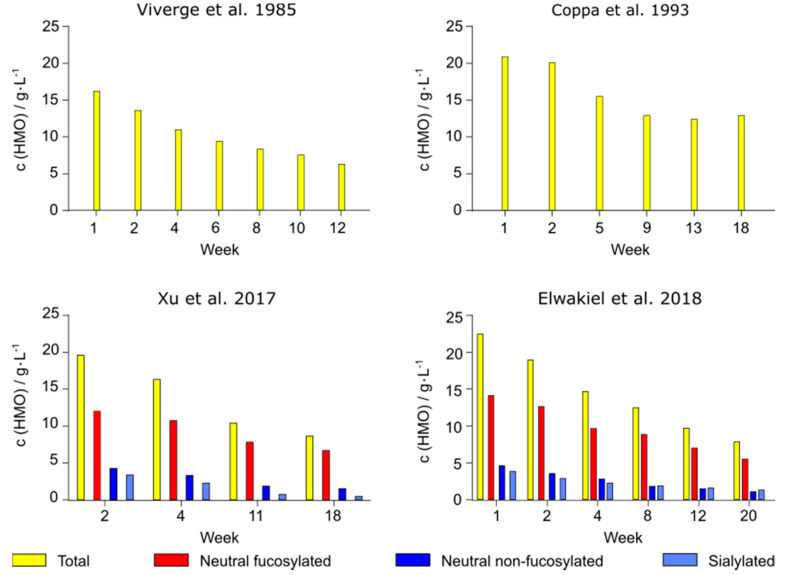
Change of the HMO content during the course of lactation. The mean content of total HMOs (yellow) decreases by up to 67% during the course of lactation, which has been shown by Viverge et al. [6] and Coppa et al. [9] (top images). Differentiation of HMOs into the three groups of neutral fucosylated, neutral non-fucosylated and sialylated HMOs shows a similar trend for the individual groups (Xu et al. [4] and Elwakiel et al. [5]). Compared to colostrum, these respectively decrease by up to 61%, 86% and 66% in mature milk.

Newborns rapidly develop during their first months of life requiring variable nutritional intake [14]. A great number of studies have analyzed the HMO concentration during the course of lactation [5,6,8,9,12,15,16,17,18,19,20,21,22,23,24,25,26,27,28,29,30,31,32,33,34,35,36,37]. However, only a few performed a quantification of the absolute HMO content (Table 1). Based on these studies, the mean concentration of total indigestible oligosaccharides decreases with progressive infant age regardless of the Secretor status (Figure 2). In colostrum (day 1–5) and transition milk (day 6–14), the sugars respectively make up 16–22 g/L [5,8] and 14–20 g/L [6,9] while in mature milk, the HMO concentration steadily decreases by about 38% to 65% compared to early milk (22 g/L vs. 8–13 g/L measured 18–20 weeks after birth [5,9]). In fact, the decline in concentration applies for all three different types of HMOs, while the lactose concentration is subject to an overall increase [6,9,31] On analysis of the three different HMO types, Xu et al. and Elwakiel et al. reported a decrease of neutral fucosylated and sialylated HMOs by 44–61% and 65–66% for the period of 18–20 weeks, while the neutral non-fucosylated HMO content declines most drastically by 77–86% (Figure 2) [4,5].

Obviously, the analysis of the absolute HMO content does not give detailed information about the change of individual HMOs with regard to different factors. To address such questions, a good number of studies have therefore focused on the quantification of the most important milk sugars [7,12,17,20,28,29,42,43,46,47,48]. These have for instance shown that 3FL increases up to almost threefold throughout the course of lactation regardless of the milk status and gestational age [7,12,17,28,29,32,43,47,48].

**Figure 3:**
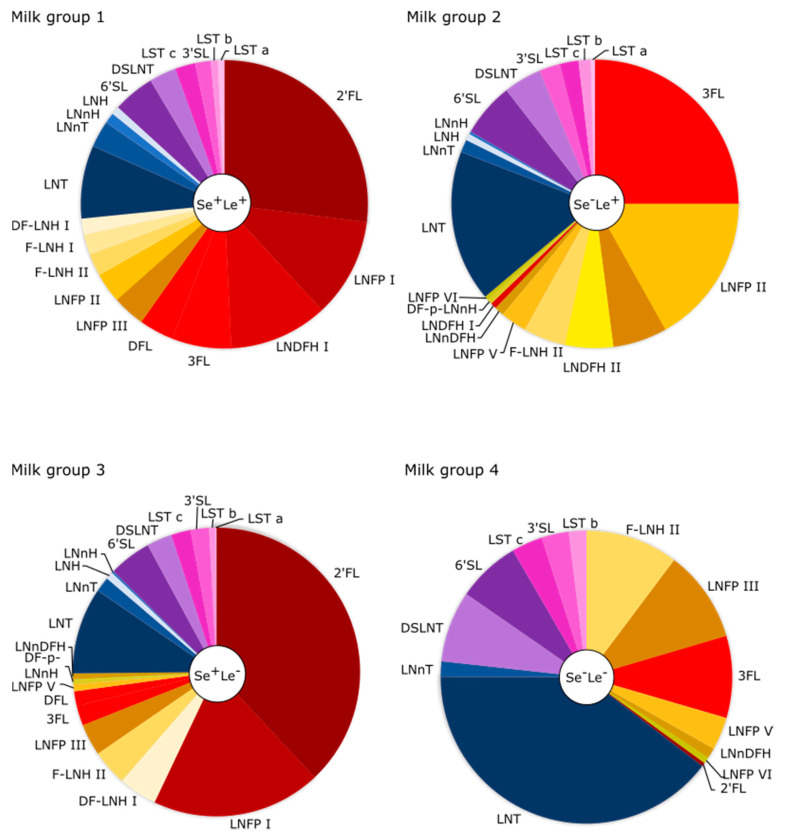
Most dominant HMOs of term-delivering mothers differentiated by the Secretor status. The ten most dominant neutral fucosylated (except for Se^−^Le^−^), the four most important neutral non-fucosylated and the six most important acidic HMOs are respectively displayed in red to light orange, blue and violet to pink.

In addition, HMOs such as 6′-sialyllactose (6′SL), difucosyllacto-N-hexaose a (DFLNHa), disialyllacto-N-tetraose (DSLNT), lacto-N-tetraose (LNT), and fucosyllacto-N-hexaose II (F-LNH II) are subject to decrease. However, unlike other HMOs whose maximum is reported in colostrum, their maximum is found in late transition (week 2) or early mature milk (week 3) [12,43]. An overview of the most dominant HMOs found in term-delivering mothers of the four different milk groups is given in Figure 3. Raw data derived from the respective studies (Supplementary Table S1) are given in Supplementary Tables S2–S5. The underlying pooled mean values were derived from the studies that differentiated mothers of the four milk groups [7,17,18,29,41,43,44,49]. Respective mean values and pooled mean values for individual HMOs of mothers delivering preterm are given in Supplementary Tables S6–S9. Note that Coppa et al. 1999 and 2011 [17,41] reported high concentrations of LNDFH II and TFLNH (>2 g/L) in milk of Italian mothers. However, as these observations have not been observed independently, the concentrations of mentioned HMOs were not taken into account in Figure 3. For further discussion on the concentrations of LNDFH II and TFLNH, the reader is referred to Thurl et al. 2017 [50] who systematically reviewed the concentration of 33 oligosaccharides and compared the milk of mothers of different gestational ages. In fact, they show that the total concentration of neutral and acidic HMOs is respectively 28% higher and 54% lower in term compared to preterm mothers. Despite the lower neutral HMO concentration in preterm mothers, the concentrations of individual HMOs such as 2′FL, DF-LNH II LNT and F-LNH-II are higher than in term mothers. Conversely, all acidic HMOs under investigation were found at higher concentrations in preterm compared to term mothers [50].

Genetic predisposition, course of lactation and gestational age predominantly define the interindividual variability of human milk sugars. However, several recent studies have identified maternal [5,12,30,37,44,51,52] and infant [34,37,44,53] characteristics, as well as mode of delivery [12,37], parity [12,37,44,54], environmental [51], geographical [19,51,54,55], regional [13,51,54] and seasonal [34,54] factors to be responsible for differences in the composition of human milk sugars.

## 2. HMOs Shaping the Neonate

Human milk oligosaccharides already circulate in the blood of pregnant mothers at least from week 10 on [56], reaching the fetus through the amniotic fluid [57] and likely through the umbilical cord passing the placenta [58].

During the first month of life, infants consume about 500–650 mL breast milk per day, which rises to around 750–800 mL after four to five months of lactation [59]. This corresponds to absolute amounts of about 7–14 g and 6–10 g, respectively (calculated from mean values given in Figure 2).

Clearly, HMOs are consumed orally, passing the upper gastrointestinal tract, and occasionally reach the nasopharynx as well as the upper respiratory tract through aspiration. The sugars are resistant to the main digestion mechanisms of the infant such as low pH in the stomach as well as salivary, pancreatic and brush boarder enzymes and thus arrive at the distal small intestine and the colon only minimally digested (<5%) [60]. In numbers, 40–50% of HMOs are excreted in the feces [61], 1–5% are absorbed into the blood stream and excreted in the urine [62,63,64], while the remaining portion is prone to microbial catabolism. However, note that the digestion of HMOs also changes during the course of lactation. Thus, it has for instance been shown that they disappear from the neonatal feces once feeding other than mother’s milk is introduced [11].

Both, intact and catabolized HMOs fulfill crucial tasks for neonatal protection and development. Most strikingly, they support and concurrently shape the neonatal immune system by various modes of action, including pathogen protection, maturation of the gut microbiome, fostering of the intestinal barrier function and maturation of immune cells. In the following, in vitro, preclinical and clinical studies demonstrating beneficial effects on the neonatal development by either human milk-derived or synthetic HMOs or HMO fractions are summarized.

### 2.1. Pathogen Protection

Pathogens gain access to the human body through various ports of entry including the mucosa of the gastrointestinal, respiratory, reproductive and uroepithelial tract as well as through the skin after injury. Physical contact between host cells and many pathogens or their toxins is mediated by carbohydrate-binding proteins. These proteins (or protein motifs) are called lectins and recognize specific moieties of surface-exposed glycans either on host or pathogen cells, which resemble HMO fragments or individual HMOs. Thus, indigestible mother milk sugars act as soluble decoy receptors that interfere with the lectin-glycan association for pathogens such as *Entaboeba histolytica* [65], *Campylobacter jejuni* [66,67], *Clostridium difficile* [68,69], enterohaemorrhagic [70], entereopathogenic [71,72,73,74,75,76,77], enterotoxic [78], uropathogenic [78,79,80] *Escherichia coli* [81], *Helicobacter pylori* [82], *L. monocytogenes* [83], *Neisseria Meningitidis* C [84], *Pseudomonas aeruginosa* [75,85,86], *Salmonella enterica* [74,75], *Staphylococcus aureus* [87], *Vibrio cholerae* [70,74,88], human immunodeficiency virus [89], influenza virus [90,91], norovirus [92,93,94] and respiratory syncytial virus [95]. Table 3 summarizes HMOs that have been shown to block pathogen adhesion in vitro. In addition, in silico studies indicate that 3′SL and 6′SL are decoy receptors for polyomavirus VP1 [96,97], but this has not been proven in vitro so far.

Besides the competitive binding activity of HMOs to lectins, a couple of studies indicate different modes of action. For instance, Gonia et al. have shown that hyphal morphogenesis of *C. albicans* is disturbed by milk oligosaccharides on the cellular and gene expression level thus inhibiting pathogen-host interactions [98]. Angeloni et al. used microarray glycoprofiling and proved that 3′SL was able to modulate the glycocalyx by reduced expression of sialyltransferases, thus likely diminishing enteropathogenic *E. coli* adhesion sites [73]. Chen et al. showed that HMO pretreatment globally alters host signaling pathways resulting in differential subcellular localizations of *L. monocytogenes* [83]. *L. monocytogene* allowing an easier pathogen clearance. [87] Jantscher-Krenn et al. and Lin et al. presented data on the cytoprotective effect, respectively, against *Entaboeba histolytica E. hystolytica* [65] and uropathogenic *E. coli* [80], which has been related to antiapoptotic and anti-inflammatory properties of HMOs [80]. Rather recent research has attributed HMOs with antimicrobial activities against specific strains of *Streptococcus agalactiae* and *Acinetobacter baumannii* as well as antibiofilm activities against strains of *Streptococcus agalactiae* and *Staphylococcus aureus*. It was hypothesized that promiscuous incorporation of HMOs into the capsular polysaccharide and peptidoglycan/glycan binding proteins of the cell walls increases the overall membrane permeability and disturbs biofilm production [87,99,100,101,102,103]. The antimicrobial activity against individual *S. agalactiae* strains was shown for LNT, LNnT [104], sialylated variants of LNT [103] and fucosylated HMOs such as LNFP I–III [104] and LNDFH I [99]. This might explain why Lewis-positive mothers and their infants are less likely to be colonized with *S. agalactiae* and why colonization clearance in these infants has a higher probability than for infants born to Lewis-negative women [99].

Some preclinical and clinical studies corroborate the protective effects of HMOs against a few pathogens displayed in Table 3. In general, higher HMO levels in mother’s milk represented by LNFP II appear to be associated with fewer respiratory and enteric problems of newborn humans [105].

A significant reduction of *C. jejuni* colonization and intestinal clearance in mice has been achieved by neutral human milk oligosaccharides and 2′FL or if dams of suckling pups have been transfected with the human α1,2-fucosyltransferase gene [66]. In addition, Yu et al. demonstrated that 2′FL was able to attenuate intestinal inflammation and induction of inflammatory signaling molecules [67]. Morrow et al. analyzed Mexican mother-infant pairs and detected a positive correlation of 2′FL and LNDFH-I concentrations and the reduction of incidence and severity of diarrhea respectively related to *C. jejuni* and caliciviruses, which include the species of noroviruses [25,106].

Moreover, preclinical and clinical data for the protection against HIV and rotavirus exist. In mice and pigs, either 2′FL or HMO mixtures containing 2′FL, LNnT, 6′SL, 3′SL and sialic acid diminished rotavirus binding, replication, diarrhea incidence, duration and severity. These were related to a reduced gene expression of viral enterotoxin [107], changes of the gut microbiome and mucosal and systemic increase of immune cells accompanied by a balanced immune response [107,108,109,110,111]. Two observational studies on African mother-infant dyads suggested a reduction of virus transmission from HIV-infected mothers to breastfed infants and a decreased mortality of HIV-exposed babies during breastfeeding. These effects respectively correlated with higher absolute concentrations of HMOs such as LNnT [112] and fucosylated oligosaccharides (2′FL, 3FL, LNFP I–III) [113].

Furthermore, inhibition of colonization of *S. pneumoniae* in the nasopharynx and lungs of rabbits as well as protection from bacteremia was achieved by LNnT and its sialylated derivatives such as 3′-sialyllacto-N-neotetraose (3′SLNnT). However, 3′SLNnT was for instance not able to protect against acute otitis media related to *S. pneumoniae*, *H. influenzae* and *M. catarrhalis* infection in an interventional clinical study [114]. Similarly, the positive effects of 3′SL to decrease and partly cure *H. pylori* colonization in rhesus monkeys [115] could not be confirmed in two human interventional studies [116,117].

Idota et al. treated the intestinal loops of rabbits with the subunit B of the *V. cholera* toxin and observed a significant inhibition of infection-associated fluid accumulation [88]. Steenhout et al. observed a significant reduction of *C. difficile* toxins A and B in infants fed 2′FL/LNnT-fortified formula compared to infants receiving formula without HMOs [118]. Protection against diarrhea related to the heat-stable toxin of *E. coli* in mice was conferred by neutral fucosylated HMOs [119]. This was revealed by data of human neonates consuming milk with a higher proportion of 2-linked fucosyloligosaccharides that had less *E. coli* toxin-associated diarrhea with reduced severity compared to those consuming milk of lower 2-linked fucosyloligosaccharides levels [120]. In addition, the total HMO fraction and 2′FL were respectively able to significantly reduce EPEC colonization [76] and guard against *E. coli* O157 in mice [121].

Among others [109,111,122], the latter study reveals that the protective function of HMOs is not only due to their structural similarity to receptor antigens. In fact, HMOs act on the gene expression of intestinal cells enhancing the intestinal barrier function, modulate intestinal and systemic immune cells controlling immune maturation and homeostasis, and are associated with establishing a probiotic gut microbiota composition [121]. These effects will be discussed in the following chapters.

### 2.2. Natural Prebiotics

The development of the intestinal flora is interconnected with metabolic and immune maturation, determining behavior and (patho-)physiology of the host [130,131].

Currently, it is under debate whether the first seed for colonization is already planted in utero, contradicting the traditional notion that this happens during birth [132,133]. In any case, prenatal and perinatal factors such as maternal diet, disease state, biodiversity of the environment, use of antibiotics, mode of delivery and feeding practices “imprint a specific hallmark” on the gut microbiota of babies [132].

Only a few bacterial families within the phyla of *Firmicutes* and *Proteobacteria* colonize the immature gastrointestinal tract of babies [134]. With progressive age, these inhabitants are displaced by *Bacteroidetes* and *Actinobacteria* leading to a microbial colonization of increased alpha diversity [135,136,137,138,139]. The early successive gut ecology is mainly dominated by *Bifidobacterium* [34,140,141,142], which can make up more than 90% in breastfed infants compared to about 50% in formula-fed infants [136,143,144]; an effect that is also observed in piglets [131,145]. In part, this is due to initial seeding before and during birth as well as maternal transmission of commensal bacteria present in mother’s milk [133,146]. However, it also indicates prebiotic mother’s milk contents. This is corroborated by the facts that fucosylated HMOs alleviate the depletion of bifidobacteria in C-section born infants [147] and that weaning accelerates microbial aging towards an adult-like composition [131] of noticeable decrease in *Bifidobacterium* [134,136,148].

The presence of a “bifidus factor” in human milk that supports the growth of commensal bacteria was postulated almost a century ago [149] and was proven to be composed of oligosaccharides [150,151,152,153,154]. A higher total HMO content [155,156] in mothers of milk groups 1, 2 and 3 compared to milk group 4 [41] as well as individual HMOs such as 2′FL [156] correlates with increased amounts of bifidobacteria in neonatal guts. In addition, infants of Secretor mothers establish earlier and more frequent bifidobacterial colonization [157], which appears to positively influence the amount of *Bifidobacterium* in children even at later ages of 2 to 3 years [158]. An intervention with HMO and 2′FL respectively showed an increase of *Actinobacteria* in fecal [159] samples and in a semi-continuous colon simulator model that was accounted for by a decrease of *Proteobacteria* in the latter study [160]. Notably, a microbiota shift towards probiotic dominance was recently achieved by feeding infants with 2′FL/LNnT-supplemented formula for 6 months [161] and even in healthy adults that received 2′FL and/or LNnT for only two weeks [162]. Taking these results together, it appears that Secretor milk is more beneficial for bifidobacterial growth than non-Secretor milk.

In order to elucidate the prebiotic molecular mechanism of HMOs, several bacteria have been tested for their ability to grow on individual or total HMOs as the sole carbon source in vitro. Mainly *Bifidobacterium* [134,141,157,163,164,165,166,167,168,169,170,171,172,173,174,175,176,177,178,179,180,181,182,183] and individual strains of *Bacteroides* [167,177], *Enterococcus* [177], *Lactobacillus* [177,184], *Streptococcus*, and *Clostridium* cluster IV/XIVa [185] are adapted to utilize oligosaccharides. Intriguingly, the extent of neonatal HMO digestion correlates with these genera [139], overall indicating that mother’s milk induces a selective pressure towards commensal gut colonization.

Among HMO utilizers, bifidobacteria co-evolved with humans and developed the most complex pangenome for the digestion of human milk oligosaccharides. This serves efficient protocooperation [141,183,186] within its own but also with other commensal genera [133,187]. In fact, their well-organized use of HMOs was corroborated by De Leoz et al. [137] and Borewicz et al. [142], who showed a negative correlation of fecal HMO concentration and, among other genera, bifidobacterial abundance. However, HMO consumption differs drastically even within the same (sub-)species, which is related to the fact that individual strains evolved a very specific genetic reservoir to fit into defined catabolic niches. Still, either an extra- or an intracellular HMO assimilation strategy can be generally described for the main bifidobacterial species of the neonatal gut, namely, *B. bifidum*, *B. breve*, *B. longum* subsp. *longum*, *B. longum* subsp. *infantis*, *B. infantis, B. pseudocatenulatum* and *B. kashiwanohense* [188]. An excellent overview of the genetic repertoire of these species was recently given by Sakanaka et al. [188]. Glycoprofiling of oligosaccharide degradation products and genomic analysis discovered that most *B. longum infantis* strains are equipped with a whole machinery of enzymes for intracellular catabolism [166,168,169,170,178,179,189]. The relevant genes are highly conserved in a cluster consisting of ATP-binding cassette transporter (ABC) molecules with solute-binding proteins (SBPs) of high HMO affinity and intracellular glycosyl hydrolases such as fucosidases, sialidases, galactosidases and N-acetylglucosaminidases. Similarly, *B. breve*, *B. pseudocatenulatum* and *B. kashiwanohense* [190] also import HMOs for intracellular degradation, but with reduced substrate bandwidth [134,175,176,179]. Furthermore, the majority of *B. breve* preferably assimilate LNnT, LNT and its degradation product lacto-N-biose (LNB) [169,170,176,179,181], a metabolic pathway that is also conserved in *B. pseudocatenulatum* [134,169] and *B. kashiwanohense* [179]. Like *B. infantis*, most *B. bifidum* bacteria are able to assimilate most HMOs, but individual strains rather rely on the extracellular assimilation strategy. *B. longum* strains are capable of disintegrating a limited number of fucosylated [176,188] but mainly non-fucosylated neutral HMOs in the extra- and intracellular space [180]. It thereby shares the possibility with *B. bifidum* of disintegrating LNT by lacto-N-biosidase, yielding lactose and LNB [169,170,180], which is taken up by both for further catabolism. Intriguingly, some *B. bifidum* strains altruistically leave extracellular HMO digestion products unconsumed for neighboring species or strains within the bifidobacterial ecosystem [141,183,191]. This cross-feeding strategy is believed to maximize nutrient exploitation to foster the dominance of *Bifidobacterium* [141].

Commensal gut bacteria whose growth is nurtured by HMOs (*Bacteroidetes*, *Enterococcus*, *Lactobacillus*, *Streptococcus*, *Clostridium* cluster IV/XIVa and mainly *Bifidobacterium*) produce several molecules that are beneficial for infants including B and K vitamins as well as bacteriocins and antivirals [133,192,193,194,195]. Moreover, their carbohydrate catabolism yields multiple metabolites among which short chain fatty acids (SCFAs) like acetate, butyrate and propionate are the most important. In fact, an increase in the number of these commensals concurrent with enhanced fecal SCFA production was achieved by total and individual HMOs such as 2′FL, 3FL and DFL in in vitro fermentation studies of infant feces [159,196,197,198]. Similar observations were made in newborn mice [199], rats [122] and pigs [108,124,145,200] supplemented with LNnt, 2′FL, a HMO mix (2′FL, LNT, LNnT, 6′SL) or total HMOs. SCFAs derived from HMOs exert numerous positive effects on the neonatal host. A detailed description of their molecular modes of action is not within the scope of this review, but key functions should be mentioned. These small organic acids contribute to the host energy metabolism, influencing several organs and providing up to 10% of total caloric requirements and up to 70% of energy for colonocytes thus supporting colonic function and health [201]. They trigger the proliferation and differentiation of colonocytes, enhance the intestinal epithelial barrier function [199] by induction of mucin 2 expression and differentiation of goblet cells, regulate and modulate immune function [202] and are thought to be the key sensing molecules of the microbiota-gut-brain axis affecting development and homeostasis of the central nervous system [194,203,204,205,206,207].

In addition, microbial degradation of HMOs not only yields SCFAs but also lactate and concurrently reduces the intestinal pH [159,197]. On the one hand, this facilitates the uptake of essential nutrients by the host. On the other hand, SCFAs are toxic to several detrimental bacteria in their nonionized form prevalent at low pH [208]. This HMO-mediated competitive exclusion of pathogens is even further promoted by milk oligosaccharides such as 3′SL and 6′SL that induce a transcriptomic and physiological response of some bifidobacterial strains for a physical contact with intestinal cells [209,210,211,212]. At this interface, commensals directly influence intestinal function and shape the architecture of the gastrointestinal immune system [211] by stimulating the maintenance of the sterile mucus layer [213], immunomodulatory cytokines, the epithelial secretion of antimicrobial peptides and the gastrointestinal lymphoid tissue [138].

This chapter revealed that crucial processes of neonatal development are tightly associated with the establishment of a healthy gut microbiota nourished by oligosaccharides from human milk. However, HMOs themselves not only act as natural prebiotics and soluble decoy receptors for pathogens. They are even able to directly affect critical physiological processes such as maturation and maintenance of the intestinal barrier function as well as immune development and homeostasis; topics that will be covered in the next section.

### 2.3. Maturation and Maintenance of the Intestinal Barrier and Function

Intestinal epithelial cells form a physical barrier in the gastrointestinal tract and are in close cooperation with commensal microbiota, immune and stromal cells to defend the body from pathogens at the forefront. Multipotent stems cells continuously differentiate from the crypt along the villus axis into nutrient-absorbing enterocytes, secretory goblet, enteroendocrine and Paneth cells as well as microfold and tuft cells that communicate with the intestinal immune system. This cellular barrier is reinforced by tight junctions as well as the glycocalyx and the mucus layer. Tight junctions control the paracellular permeability of the epithelial surface, while the glycocalyx, a complex meshwork of glycolipids and glycoproteins, is responsible for microbial adhesion, recognition, communication and toxin repulsion on the apical side. The mucus covers the inner surface of the gastrointestinal tract and provides a barrier function against pathogens and exogenous molecules [214,215].

Direct modulatory effects of HMOs on intestinal cells and structures are sparse. However, 2′FL supplementation was shown to increase villus areas and heights [122], crypt depths [216], elevated expression of brush boarder enzymes [124], rodents [122,216] or pigs [124], suggesting supporting effects for intestinal absorption. In vitro studies with non-transformed fetal crypt cells, preconfluent and postconfluent absorptive and secretory intestinal cells detected HMO-mediated cell maturation from the crypt to the villus [217,218,219,220]. Representatives of fucosylated, neutral non-fucosylated and sialylated HMOs inhibited the proliferation of immature secretory crypt cells through cell cycle arrest [218,219,221] or apoptosis [218,219,220,221] and enhanced their differentiation in the presence of 2′FL [219] and 6′SL [217]. In contrast, tested HMOs and LNnT respectively increased the nutrient absorption and barrier function of mature absorptive villal cells [219,220]. As mentioned above, Angeloni et al. detected that 3′SL is able to reduce the expression of sialyltransferases in intestinal cells thus modulating their glycocalyx for reduced enteropathogenic *E. coli* adherence [73]. Kong et al. proved that 2′FL and 3FL enhance the stability of the glycocalyx and its barrier function and integrity [222]. The same two HMOs induce the transcription of mucins (MUC2) in goblet cells [223], which was even confirmed in HMO-supplemented mice [224]. Furthermore, elevated mucin expression concurrent with reduced pathogenic adhesion and membrane permeability in 2′FL [121] or 3′SL-supplemented rats [225] as well as an increase of the mucosal proportion in 2′FL-supplemented pigs challenged with *E. coli* is in agreement with these observations [124]. Finally, Chleilat et al. reported that HMOs promote reduced intestinal permeability in female rats related to increased mRNA levels of tight junction proteins such as zonula occludens 1 and occludin [225].

### 2.4. Intestinal Immune Development and Homeostasis

Intrauterine and perinatal mucosal and systemic immunity deviates from mature immunity characterized by a propensity towards Th2 helper cells dominated by anti-inflammatory cytokine production. Rapidly after birth, the proportions of pro-inflammatory Th1 and regulatory T cells increase to achieve Th1 and TH17 homeostasis with Th2 in order to tolerate commensal colonization while protecting against extracellular and intracellular antigenic substances or pathogens [132,226]. HMOs have thereby been ascribed to mediate this “vigilant establishment” [227] by interaction with intestinal and immune cell receptor molecules such as C-type lectins [228,229], galectins [230], selectins [231], siglecs (sialic acid-binding Ig-like lectins) [228,232] and toll-like receptors [229,233,234,235].

In the gut, the intestinal epithelium is in steady concert with mucosal immune cells of the Peyer’s patches and the mesenteric lymph nodes to guarantee an appropriate response to multifaceted external stimuli and protect against overshooting reactions. At this forefront of host defense, milk sugars attenuate mucosal expression of inflammatory cytokine and take care of a balanced immune response as was evaluated in vitro and in vivo [236].

For instance, 3′SL-induced peroxisome proliferator-activated receptor γ (PPARγ) in Caco-2 cells that regulates peptidoglycan recognition protein 3, which in turn reduced inflammation (NFκB ↓; IL-8 ↓, IL-12 ↓, TNF α ↓, with ↓ meaning reduced expression) [237]. Later, Zehra et al. confirmed the PPARy-mediated anti-inflammatory action of sialylated HMOs with 6′SL showing IL-8 and MIP-3α reduction upon pro-inflammatory stimuli related to reduced AP1 and NFκB activity. In contrast, the same study showed PPARy independent immune attenuation by 2′FL [238]. Furthermore, active mimicry of inflammation [200], presence of pathogen-associated molecular patterns [234] as well as *C. jejuni* [67], *E. coli* [239] or *Salmonella* [240] were also attenuated by a reduced expression of inflammatory markers (IL-1β, IL-8, I L-12, MCP-1/2, MIP-3α) in the presence of specific colostral HMOs like 2′galactosyloliogosaccharide (2′GL) as well as total and individual HMOs like 2′FL, 3FL, 3′SL, 6′SL and LNFP I [67,234,239,240]. In one of these studies, elevated cytokines involved in tissue repair and Th1/Th2 homeostasis were detected [234]. Furthermore, exposure of intestinal epithelial cells to bacterial DNA boosted inflammatory Th1 IFNγ and regulatory IL-10, reduced Th2 type IL-13 and induced galectin release that attracts migratory dendritic cells. Intriguingly, intestinal cells imprinted with this pathogenic stimulus instructed naïve T helper cells towards a similar response (IFNγ, IL10) via dendritic cells suggesting a HMO-dependent development of adaptive immune response [241].

Several preclinical studies corroborated the effects of HMOs observed for intestinal cells in vitro and indicate that milk sugars promote lymphocyte proliferation (B cell, regulatory T cells, Th1 cells) [242] and Th1/Th2 balancing in the mucosa. In a murine model of chronic colitis, 3′SL resembling some pathogen surface structures induced Th1/ Th17-dependent inflammation via dendritic cells of the mesenteric lymph nodes potentially educating the infant’s immune system about foreign antigens [243]. In pigs, HMO supplementation decreased pro-inflammatory IL-8 expression, increased Th1 cytokines (IL-12, INF-γ) balanced by regulatory IL-10 while attenuating the immune response to rotavirus infection [108]. Likewise, the intestinal inflammation in mice caused by *C. jejuni* attenuated an inflammatory response on the gene expression and protein level (IL-1β ↓, TNF α ↓, IL-6 ↓, MIP-2 ↓), while unchanged IL-17 levels indicated no induction of adaptive immune response [67]. Evidence that 2′FL exhibits positive immunomodulation was further shown in healthy and rotavirus infected rats. Both increased intestinal toll-like receptor expression upon supplementation likely facilitating an improved communication with the mucosal immune system as well as viral clearance [111]. Furthermore, in a mouse model of food allergy, 2′FL and 6′SL reduced allergy symptoms and proved attenuative modes of action reducing mast cell numbers and inhibiting their degranulation while increasing regulatory T cells (CD4^+^, CD25^+^, IL-10^+^) in Peyer’s patches and mesenteric lymph nodes [244]. Overall, the presented studies indicate supportive functions of HMOs achieving immune balance upon pathogen stimuli. However, note that there are some conflicting data. Hester et al. detected no effect of HMOs on intestinal Th1/Th2 homeostasis [107], which is in agreement with data from 2′FL-supplemented healthy rats exhibiting a parallel reduction of pro- and anti-inflammatory mucosal cytokines without tendency towards a Th1 increase [122].

The etiology of chronic inflammatory diseases of the gastrointestinal tract like colitis ulcerosa and necrotizing enterocolitis (NEC) are not well understood but are likely related to dysbiosis. NEC is a devastating disorder that mainly affects preterm infants of very low birth weight. Comparison of breast-fed and formula-fed infants indicated a lower incidence of NEC related to HMOs, which was corroborated by the fact that children of mothers with a low Secretor phenotype [245], low HMO diversity [246], low LNDFH I [246] but mainly DSLNT [246,247] concentration are more prone to the disease. In fact, the most dominant sialylated HMO of mothers delivering preterm infants is DSLNT as shown in Supplementary Tables S6–S9. These observational results were underlined by interventional studies with HMOs in rodents [248,249,250,251]. In addition, 2′FL and 3FL [224,252] but not 3′SL or 6′SL fostered [253] mice are more resistant to DSS-induced colitis of reduced severity associated with decreased markers of inflammation, histological and diarrhea scores.

Even though observed effects have been mainly linked to the prebiotic effects of HMOs, their immune attenuation and support of the intestinal barrier might play a role as well. This was proven in murine NEC models, which exhibited a decrease of pro-inflammatory markers [250,254], preservation of the intestinal mucosal architecture by gavage of 2′FL [250] and preservation of mucin-expressing goblet cells by the gavage of total HMOs [224]. In an interventional study in pigs, neither a reduction of NEC lesions nor an anti-inflammatory effect could be achieved by a mixture of four HMOs or more than 25 HMOs. Thus, further studies appear to be necessary to clarify the roles of HMOs in the development of NEC [200].

### 2.5. Systemic Effects

About 1–5% [62,63,64] of ingested HMOs are absorbed into the blood stream where they can exert balancing effects on the systemic immune system. This became evident on analysis of peripheral blood mononuclear cells (PBMCs) that were isolated from breastfed and formula-fed infants either receiving formula with or without 2′FL. Notably, 2′FL-supplemented babies showed similar anti-inflammatory cytokine levels as breastfed ones, which were ~30% to ~80% lower than in infants receiving no 2′FL [255]. Moreover, 2′FL and a mix of 4 HMOs (2′FL, LNnT, 3′SL, 6′SL) respectively increased splenic lymphocytes in mice [256] and the population of PMBCs in piglets, while increasing Th1 IFNγ in both cases [109]. In fact, in vitro studies on PBMCs including dendritic cells, lymphocytes and macrophages corroborated the observation that HMOs directly interact with systemic immune cells and affect their proliferation, differentiation and maturation. Xiao et al. have shown that total HMOs promote immune tolerance by acting on DCs that communicate with T cells through the presentation of antigens. The presence of milk oligosaccharides induced maturation of DCs, elevated regulatory IL-10 and IL-27 as well as pro-inflammatory IL6 but not TNF-α and caused the differentiation of naïve to regulatory T cells. Furthermore, the homeostatic function of HMOs via DCs upon lipopolysaccharide (LPS) stimulation was expressed by the release of IL-10 and IL-27, the reduction of pro-inflammatory markers (IL-12p70, IL-6 and TNF-α), an increase of regulatory T cells and a decrease of the Th1 frequency [229].

Data supporting HMO-mediated immune balancing via dendritic cells were obtained in non-obese diabetic mice, which are characterized by a Th1/Th17 polarized immune response. Here, HMOs induced anti-diabetogenic cytokine levels and the differentiation of dendritic cells towards a regulatory/tolerogenic phenotype thus reducing the incidence of diabetes and pancreatic insulitis later in life. While these positive effects were related to an alleviation of microbial dysbiosis and SCFAs in parallel, in vitro studies underlined that HMOs are even solely able to modulate blood mononuclear dendritic cells towards a regulatory cytokine microenvironment triggering an increase of regulatory T cells [257]. However, as 2′FL and 6′SL have not been able to modulate human dendritic cells, further studies are necessary to discover the active single sugars [258].

Studies using cord blood mononuclear cells (CBMCs) and PBMCs further reveal Th1/Th2 balancing of the neonate by acidic HMOs while increasing the regulatory T cell population [259,260]. In addition, fucosylated HMOs support regulatory immune response by inhibition of IFN-γ and IL-12 production [261], reduction of cell proliferation and IL-10 expression under non-stimulating conditions [261,262]. Upon respiratory syncytial virus infection, PBMCs have an anti-inflammatory effect in the presence of milk oligosaccharides [95]. In contrast, HMOs tend to potentiate the immune response upon co-stimulation with LPS and phytohemagglutinin by increasing immune cell proliferation and inflammatory TNF-α as well as decreasing T-cell population [262].

Direct immune modulatory effects on macrophages have also been shown for neutral HMOs [261,263,264]. These increase the production of prostaglandins as well as the expression of nitric oxide synthase, cyclooxygenase, pro-inflammatory but also regulatory cytokines [235,263,264]. Aside from this, Atochina et al. [263] discovered that LNFP III-stimulated macrophages activate natural killer cells. Finally, Bode et al. indicated that acidic HMOs protect tissues from leukocyte (monocyte, lymphocyte and neutrophil) infiltration [265]. This is an initial key event of inflammatory diseases like NEC, whereas neutrophil activation through formation of platelet-neutrophil complexes is crucial for progression [266]. Both events can be hindered by systemically circulating HMOs that bind to selectin surface receptors necessary for molecular docking [267].

Little evidence exists that individual HMOs are able to interact with the enteric and central nervous systems without relation to their prebiotic role in the microbiota gut-brain axis as was proposed by Tarr et al. and Jacobi et al. [203,204]. For instance, Bienenstock et al. verified that 2′FL and 3FL diminish colon motor contractions potentially by stimulating enteric neurons that in turn affect migrating motor complexes [268]. Data of Krug et al. [269], Matthies et al. [270] and Vázquez et al. [271] indicate that fucosyllactose improves long-term potentiation [269,270] and learning capabilities [271]. These were respectively assessed on hippocampal slices of rats [270], freely moving rats receiving intrahippocampal 2′FL [269] and rats that orally ingested 2′FL [271]. Among other speculated underlying mechanisms [269,270], 2′FL increased the expression of proteins in the hippocamus, stratium and frontal cortex that are fundamental for synaptic function related to learning and memory formation [271]. These molecular mechanisms were partly corroborated by Wu et al., who even attributed neuroprotective abilities to 2′FL [272], even though relations to prebiotic effects were not excluded. Observational human studies have also linked breastfeeding [273,274,275,276] and 2′FL to a better cognitive development of infants [276]. However, more studies are required to understand the modes of action in greater depth and to differentiate between HMOs that act on the brain either directly or via the microbiota-gut-brain axis.

## 3. Conclusions

Mother Nature went to great efforts to evolve the molecular class of human milk oligosaccharides, which contains a large variety of sugar molecules that are composed of five building blocks only. Their composition in human milk changes in interplay of the mother and her offspring and on demand of the growing infant to fulfill critical tasks for neonatal development and protection. This review collected in vitro, preclinical and clinical studies to elucidate that HMOs not only serve as soluble decoy receptors for pathogen protection. In fact, they actively shape immature gut microbiota, develop the intestinal and systemic immune systems, while supporting the function of the enteric and central nervous systems.

The recent advent of chemical and biotechnological production techniques has made several individual HMOs such as 2′FL, 3FL, DFL, LNT, LNnt, 3′SL and 6′SL available for research and mothers that (must) rely on formula feeding. In the future, this will allow larger (interventional) studies to further dig into the molecular modes of actions of HMOs and will hopefully trigger the industrial production of a greater variety of such promotive sugars to serve the market with personalized infant formula.

## Figures and Tables

**Table 1:** Studies that quantified absolute human milk oligosaccharide concentrations.

HMO Content/g∙L^−1^	Period of Observation/d	Location of Study	*n* (mothers)	Reference
3.5–18.5	1–90	France	N/A	[6]
15.2–16.7	2–7	France	15	[8]
12.9–20.9	4–120	Italy	46	[9]
7.9	2–28	Germany	10	[1]
8.6–19.6	10–120	USA	45	[4]
5.3–6.5	180	Malawi	88	[4]
7.8–22.4	1–140	China	30	[5]

**Table 2:** Secretor and Lewis genetic predisposition of different populations. Note that not all studies differentiated between all groups.

Se^+^Le^+^/%	Se^−^Le^+^/%	Se^+^Le^−^/%	Se^−^Le^−^/%	Location of Study	*n* (Mothers/Children)	Reference
69	20.0	9.0	1.0	Germany	50	[38,39]
71.7	24.5	3.8		Burkina Faso	53	[40]
73.3	23.3	3.3		Italy	50	[40]
73.0	17.0	10.0		Germany	30	[29]
45.0	10.0	28.0	26.0	Gambia	60	[3]
55.6	28.6	11.1	4.8	Italy	63	[41]
46.9	34.4	18.8		Spain	32	[42]
73.0	20.0	7.0	-	China	30	[5]
67.0	24.0	5.0	4.0	Vietnam	141	[13]
75.0	19.0	4.0	2.0	Switzerland	25	[43]
70.0	20.0	7.0	3.0	Switzerland	290	[12]
75.6	11.5	11.5	1.3	Brazil	78	[44]
76.7	17.2	4.3	1.7	China	116	[37]
77.0	7.0	13.0	3.0	Germany	60	[45]
58.3	20.8	16.7	4.2	China	24	[46]

**Table 3:** Effects of HMOs on different pathogens. If not stated differently, the antiadhesive activity of HMOs describes the competitive interference of lectin-glycan association.

Pathogen	Strain/Subtype	HMO/HMO Fraction	Activity	Reference
*Aspergillus fumigatus*	CBS 113.26	3′SL	Antiadhesive	[123]
*Candida albicans*	SC5314	Total HMO	Cellular and gene expression of hyphal morphogenesis	[98]
*Entamoeba histolytica*	HM-1:IMSS	Total HMO, neutral non-fucosylated HMO, LNT, LNFP II, LNFP III	Antiadhesive, cytoprotective	[65]
*Acinetobacter baumannii*	ATCC 19606	Total HMO	Antimicrobial	[87]
*Campylobacter jejuni*	81–176, 287ip, 84sp, 166ip, 10sp, 57sp	Total HMO, 2′FL	Invasion protection, antiadhesive	[66,67]
*Clostridium difficile*	N/A	Total HMO, neutral non-fucosylated HMO, 8 individual HMOs	TcdA toxin inhibition	[68,69]
*Clostridium difficile*	N/A	11 individual HMOs	TcdB toxin inhibition	[68]
*Escherichia coli*	O18:K1	3′SL	Antiadhesive	[81]
*Enterohemorrhagic Escherichia coli*	N/A	20 individual HMOs	HLT, Stx1, Stx2 toxin inhibition	[70]
*Enteropathogenic Escherichia coli*	O119, E2348/69, 01163, 0111:H2, 01736, 0119:H6, 851/71, O142:H6	Total HMO, neutral HMO, fucosylated HMO, acidic HMO, 2′FL, 3FL, DFL, LNFP I, LNFP II, LNFP III, LNT, LNnT, 3′SL, 6′SL	Sta toxin inhibition, antiadhesive	[71,72,73,74,75,76,77]
*Enterotoxic Escherichia coli*	H4 CFA/I, 23 CFA/II, F18	Acidic HMO, 2′FL, 3′SL, 6′SL, DSLNT, LST a, 3′SL, 3FL	Antiadhesive	[78,124]
*Uropathogenic Escherichia coli*	CTF073, FVL 25 Fimbria P-like	Total HMO, neutral, tri-, penta-, high molecular weight HMO, sialylated HMO, 3′SL, 6′SL, DSLNT, LST a, 3′SL3FL	Antiadhesive, antiinvasive, cytoprotective	[78,79,80]
*Pseudomonas aeruginosa*	DSM1707	2′FL, 3FL, LNFP II, LNnFP V, LNnDFH II, 3′SL	Antiadhesive	[75,85,86]
*Helicobacter pylori*	1832, CP22, 1351,	3′SL	Antiadhesive	[82]
*L. monocytogenes*	EGD-e	Total HMO	Antiadhesive	[83]
*Neissereria Meningitidis C*	8013	Acidic and neutral HMO	Antiadhesive	[84]
*Salmonella enterica*	Serovar fyris	Acidic, neutral low weight HMO, 6′SL, 2′FL, 3FL	Antiadhesive	[74,75]
*Staphylococcuss aureus*	USA300	Total HMO	Antimicrobial, antibiofilm	[87,125]
*Streptococcus agalactiae*	CNCTC 10/84, GB590, GB2, ST III (COH-1, A909, NCTC)	Total HMO, neutral HMO, fucosylated HMO, LNT, LNnT, DFL, LNFP I, II, III, LNnH, LNDFHI, LST a, LST c, DSLNT, 3′SL, 6′S	Antimicrobial, antibiofilm	[87,99,100,101,102,103]
*Streptococcus pneumoniae*	R6, SIII, AII	LNT, LNnT, 3′SL, 6′SL, LSTc, 3′SLNnT, 3′GL	Antiadhesive	[126,127,128]
*Vibrio cholerae*	N/A	Acidic HMO, neutral high. mol. weight HMO, 18 individual neutral and sialylated HMOs, 3′SL	Antiadhesive, cholera toxin B inhibition	[70,74,88]
Influenza virus	H1N1, H1Nx, H9N2	3′SL, 6′SL	Antiadhesive	[90,91,95]
Human immunodeficiency virus	N/A	Total HMO	Antiadhesive	[89]
Norovirus	G.I, GII.4, GII.10, G.17	2′FL, 3FL, LNFP I	Antiadhesive	[92,93,94]
Respiratory syncytial virus	NM232	2′FL, 3′SL, LNnT	Antiadhesive	[95]
Rotavirus	OSU; G1P, G2P	3FL, 3′SL, 6′SL	Antiadhesive	[107,129]

## Supplementary Materials

Supplementary information are available online at https://www.mdpi.com/2072-6643/12/11/3568/s1. Table S1: Study information, HMO breastmilk content of term-delivering mothers, Table S2: Mean and pooled mean values of individual HMOs for mothers delivering at term belonging to milk group 1 (Se^+^Le^+^), Table S3: Mean and pooled mean values of individual HMOs for mothers delivering at term belonging to milk group 2 (Se^−^Le^+^), Table S4: Mean and pooled mean values of individual HMOs for mothers delivering at term belonging to milk group 3 (Se^+^Le^−^), Table S5: Mean and pooled mean values of individual HMOs for mothers delivering at term belonging to milk group 4 (Se^−^Le^−^), Table S6: Mean and pooled mean values of individual HMOs for mothers delivering preterm belonging to milk group 1 (Se^+^Le^+^), Table S7: Mean and pooled mean values of individual HMOs for mothers delivering preterm belonging to milk group 2 (Se^−^Le^+^), Table S8: Mean and pooled mean values of individual HMOs for mothers delivering preterm belonging to milk group 3 (Se^+^Le^−^), Table S9: Mean and pooled mean values of individual HMOs for mothers delivering preterm belonging to milk group 4 (Se^−^Le^−^).
