# Supplementary material for: In Love with Shaping You—Influential Factors on the Breast Milk Content of Human Milk Oligosaccharides and Their Decisive Roles for Neonatal Development
Source: Nutrients. 2020 Nov 20;12(11):3568. doi: 10.3390/nu12113568 (PMC7699834; doi:10.3390/nu12113568)
Supplement: Supplementary file 1 [file nutrients-12-03568-s001.pdf]

Supplementary Information

In Love with Shaping You – Influential Factors on the Breast Milk Content of Human Milk Oligosaccharides and Their Decisive Roles for Neonatal Development

Christian Hundshammer * and Oliver Minge

Wacker Chemie AG, Biosolutions, Hanns-Seidel-Platz 4, 81737 München, Germany; oliver.minge@wacker.com
* Correspondence: christian.hundshammer@wacker.com

Term-Delivering Mothers

Studies and related information reporting individual human milk oligosaccharide concentrations of mothers delivering children at term are listed in Supplementary Table S1. Reported mean values of mothers belonging to milk groups 1, 2, 3 and 4 are respectively given in Supplementary Tables S2, S3, S4 and S5. Pooled mean values ($c_{\text{pooled mean}}$) that were used for Figure 3 in the main manuscript were derived according to the following equation:

$$c(pooled\ mean)_{i,j} = \frac{c_{mean}(study)_i \cdot n_{samples}(study)_i + \cdots + c_{mean}(study)_j \cdot n_{samples}(study_j)}{n_{samples}(study_i) + \cdots + n_{samples}(study_j)}$$

with $c_{\text{mean}}(\text{study})_{i,j}$ being the reported mean value of an individual human milk oligosaccharide of a study and $n_{\text{samples}}(\text{study})_{i,j}$ being the number of samples that were analyzed to obtain the respective mean value.

Note that Coppa et al. 1999 and 2011 [17,41] reported high concentrations of LNDFH II and TFLNH (> 2 g/L) in milk of Italian mothers. However, as these observations have not been observed independently, the concentrations of mentioned HMOs were not taken into account **for Figure 3 in** the main manuscript. For further discussion on the concentrations of LNDFH II and TFLNH, the reader is referred to Thurl et al. 2017 [50] who systematically reviewed the concentration of 33 oligosaccharides and compared the milk of mothers of different gestational ages. The pooled mean concentrations of the ten most dominant neutral fucosylated HMOs are highlighted in red (if applicable). The pooled mean concentrations of the four most dominant neutral non-fucosylated HMOs are highlighted in blue (if applicable). The pooled mean concentrations of the six most dominant sialylated HMOs are highlighted in violet (if applicable).

Supplementary Table S1: Study information, HMO breastmilk content of term-delivering mothers**.**

	Coppa et al. 1999	Thurl et al. 2010	Coppa et al. 2011	Olivares et al. 2015	Tonon et al. 2019	Austin et al. 2019	Samuel et al. 2019	Absolute
n (mothers)	18	30	39	12	79	34	290	502
Duration / days	4 – 90	2 – 96	3 – 5	30	17 – 76	0 – 56	0 – 120	-
Study location	Italy	Germany	Italy	Spain	Brazil	Switzerland	Switzerland	-
Reference	[17]	[29]	[41]	[49]	[44]	[43]	[12]	-

Supplementary Table S2: Mean and pooled mean values of individual HMOs for mothers delivering at term belonging to milk group 1 ($Se^{+}Le^{+}$).
Note that values marked with * could not be reproduced by other independent studies and / or were obtained from a few measurements only. Therefore, no pooled mean values were calculated for these HMOs.

	Coppa et al. 1999		Thurl et al. 2010		Coppa et al. 2011		Olivares et al. 2015		Tonon et al. 2019		Austin et al. 2019		Samuel et al. 2019			
**HMO**	c_{mean} / **g/L**	***n*** **(samples)**	c_{mean} / **g/L**	***n*** **(samples)**	c_{mean} / **g/L**	***n*** **(samples)**	c_{mean} / **g/L**	***n*** **(samples)**	c_{mean} / **g/L**	***n*** **(samples)**	c_{mean} / **g/L**	***n*** **(samples)**	c_{mean} / **g/L**	***n*** **(samples)**	n_{total} **(samples)**	***c*** **(pooled mean) / g/L**
2′FL	2.81	90	3.13	109	2.56	10	-	-	2.20	59	2.55	164	2.38	1053	1485	2.47
LNFP I	1.21	90	1.58	109	1.18	10	-	-	0.73	59	1.26	164	0.92	1035	1467	1.02
LNDFH I	0.82	90	1.27	109	-	-	-	-	1.22	59	1.17	164	0.98	1037	1465	1.02
DFLNnH	0.42	90	0.42	109	0.36	10	0.76	6	0.53	59	0.43	164	0.68	1072	1504	0.61
3FL	-	-	0.41	109	-	-			-	-	0.34	164	0.36	1040	1319	0.36
LNFP III	-	-	0.38	109	-	-	0.12	6	-	-	0.30	164	0.34	1071	1344	0.34
LNFP II	0.36	90	0.22	109	0.45	10			-	-	0.33	164	0.31	1052	1431	0.31
F-LNH II	0.53	90	0.16	109	0.45	10	0.27	6	-	-	0.27	164	0.20	1039	1412	0.23
DFL	-	-	0.21	109	-	-	-	-	-	-	-	-	-	-	109	0.21
DFLNH I	-	-	0.33	109	-	-	-	-	-	-	0.19	164	0.14	843	1116	0.16
LNDFH II	0.20	90	0.19	109	0.20	-	-	-	0.02	59	-	-	-	-	258	0.15
LNFP V	-	-	-	-	-	-	-	-	-	-	0.04	164	0.06	851	1015	0.06
DF-LNnH	-	-	-	-	-	-	-	-			0.03	164	0.06	369	533	0.05
DF-p-LNnH	-	-	-	-	-	-	-	-	0.04	59	-	-	-	-	59	0.04
LNFP VI	-	-	-	-	-	-	-	-	-	-	0.01	164	0.03	209	373	0.02
TF-LNH	2.82*	90	-	-	2.95*	10	-	-	-	-	-	-	-	-	-	-
DFLNH II	2.36*	90	-	-	2.01*	10	-	-	-	-	-	-	-	-	-	-
DF-LNnH	0.54*	90	-	-	-	-	-	-	-	-	-	-	-	-	-	-
-LNT	1.02	90	-	-	-	-	1.23	6	-	-	0.93	164	0.70	1071	1331	0.75
LNnT	1.52	90	-	-	-	-	-	-	-	-	0.20	164	0.18	1059	1313	0.28
LNH	0.16	90	-	-	-	-	-	-	0.04	59	-	-	-	-	149	0.11
LNnH	0.09	90	-	-	-	-	-	-	0.08	59	-	-	-	-	149	0.08
6'GL	-	-	-	-	-	-	-	-	-	-	0.04	164	0.04	1051	1215	0.04
A-tetrasacch.	-	-	-	-	-	-	-	-	-	-	0.03	164	-	-	164	0.03
3‘GL	-	-	-	-	-	-	-	-	-	-	0.01	164	-	-	164	0.01
6′-SL	0.42	90	1.22	109	-	-	-	-	0.37	59	0.43	164	0.35	1071	1493	0.43
DSLNT	0.70	90	0.34	109	-	-	-	-	-	-	0.29	164	0.24	1064	1427	0.28
LST c	0.41	90	0.29	-	-	-	-	-	0.16	59	0.27	164	0.18	1072	1385	0.21
3′-SL	0.10	90	0.27	109	-	-	-	-	0.18	59	0.15	164	0.15	1072	1494	0.16
LST b	0.21	90	0.08	109	-	-	-	-	0.07	59	0.07	164	0.06	1057	1479	0.07
LST a	0.14	90	0.04	109	-	-	-	-	0.01	59	-	-	-	-	258	0.07

Supplementary Table S3: Mean and pooled mean values of individual HMOs for mothers delivering at term belonging to milk group 2 ($Se^{-}Le^{+}$).
Note that values marked with * could not be reproduced by other independent studies and / or were obtained from a few measurements only. Therefore, no pooled mean values were calculated for these HMOs.

	Thurl et al. 2010		Coppa et al. 2011		Olivares et al. 2015		Tonon et al. 2019		Austin et al. 2019		Samuel et al. 2019			
**HMO**	c_{mean} / **g/L**	***n*** **(samples)**	c_{mean} / **g/L**	***n*** **(samples)**	c_{mean} / **g/L**	***n*** **(samples)**	c_{mean} / **g/L**	***n*** **(samples)**	c_{mean} / **g/L**	***n*** **(samples)**	c_{mean} / **g/L**	***n*** **(samples)**	n_{total} **(samples)**	***c*** **(pooled mean) / g/L**
3FL	1.79	28	0.40	19	0.62	5	1.62	9	1.29	40	1.84	259	355	1.69
LNFP II	1.25	28	0.48	19	-	-	-	-	1.18	40	1.18	258	350	1.14
LNFP III	0.38	28	-	-	-	-	-	-	0.40	40	0.42	259	327	0.41
LNDFH II	0.45	28	0.23	19	-	-	0.41	9	-	-	-	-	56	0.37
F-LNH II	0.35	28			-	-	-	-	0.50	40	0.29	255	323	0.32
LNFP V	-	-	-	-	-	-	-	-	0.16	40	0.21	257	297	0.20
LNnDFH	-	-	-	-	-	-	-	-	0.02	40	0.10	78	118	0.07
LNDFH I	-	-	-	-	-	-	-	-	0.01	10	0.11	6	16	0.05
DF-para-LNnH	-	-	-	-	-	-	0.04	9	-	-	-	-	9	0.04
LNFP VI	-	-	-	-	-	-	-	-	0.02	40	0.03	91	131	0.03
2′FL	-	-	-	-	-	-	-	-	0.01	40	0.08	17	57	0.03
LNFP-I	-	-	-	-	-	-	-	-	-	-	1.51*	2	-	-
DFLNH I	-	-	-	-	-	-	-	-	-	-	0.12*	2	-	-
DFL	-	-	-	-	-	-	-	-	-	-	0.09*	1	-	-
LNT	-	-	-	-	1.22	5	-	-	1.41	40	1.11	257	302	1.15
LNnT	-	-	-	-	-	-	-	-	0.12	40	0.09	227	267	0.10
LNH	-	-	-	-	-	-	0.05	9	-	-	-	-	9	0.05
LNnH	-	-	-	-	-	-	0.02	9	-	-	-	-	9	0.02
6’GL	-	-	-	-	-	-	-	-	0.01	40	0.05	253	293	0.04
3‘GL	-	-	-	-	-	-	-	-	0.04	40	-	-	40	0.04
A-tetrasacch.	-	-	-	-	-	-	-	-	0.01	10	-	-	10	0.01
6′SL	1.14	28	-	-	-	-	0.39	9	0.35	40	0.34	258	335	0.41
DSLNT	0.42	28	-	-	-	-			0.23	40	0.28	259	327	0.29
3′SL	0.24	28	-	-	-	-	0.18	9	0.12	40	0.16	259	336	0.16
LST c	0.21	28	-	-	-	-	0.13	9	0.17	40	0.13	259	336	0.14
LST b	0.11	28	-	-	-	-	0.10	9	0.08	40	0.09	256	333	0.09
LST a	0.04	28	-	-	-	-	0.01	9	-	-	-	-	37	0.03

Supplementary Table S4: Mean and pooled mean values of individual HMOs for mothers delivering at term belonging to milk group 3 (Se^{+}Le^{-}).
Note that values marked with * could not be reproduced by other independent studies and / or were obtained from a few measurements only. Therefore, no pooled mean values were calculated for these HMOs.

	Coppa et al. 2011		Tonon et al. 2019		Austin et al. 2019		Samuel et al. 2019			
**HMO**	c_{mean} / **g/L**	***n*** **(samples)**	c_{mean} / **g/L**	***n*** **(samples)**	c_{mean} / **g/L**	***n*** **(samples)**	c_{mean} / **g/L**	***n*** **(samples)**	n_{total} **(samples)**	***c*** **(pooled mean) / g/L**
2′-FL	2.66	6	3.43	9	4.50	8	3.15	102	125	3.23
LNFP I	1.25	6	2.03	9	1.73	8	1.60	98	121	1.62
DFLNH I	-		-	-	0.61	8	0.35	93	101	0.37
F-LNH II	0.43	6	-	-	0.46	8	0.33	102	116	0.34
LNFP III	-	-	-	-	0.24	8	0.31	99	107	0.30
3FL	0.41	6	0.31	9	0.09	8	0.17	98	121	0.19
DFL	-	-	-	-	0.12	8	0.14	89	97	0.14
LNFP V	-	-	-	-	0.03	1	0.07	46	47	0.07
DF-para-LNnH	-	-	0.05	9	-	-	-	-	9	0.05
LNnDFH	-	-			0.03	3	0.06	24	27	0.05
LNDFH I	-	-	-	-	-	-	0.96*	3	-	-
LNFP VI	-	-	-	-	-	-	0.04*	4	-	-
LNT	-	-	-	-	0.44	8	0.85	104	112	0.82
LNnT	-	-	-	-	0.15	8	0.14	101	109	0.14
LNH	-	-	0.07	9	-	-	-	-	9	0.07
LNnH	-	-	0.02	9	-	-	-	-	9	0.02
6’GL	-	-	-	-	0.04	8	0.05	102	110	0.05
A-tetrasacch.	-	-	-	-	0.04	8	-	-	8	0.04
3‘GL	-	-	-	-	0.01*	1	-	-	-	-
6′SL	-	-	0.17	9	0.41	8	0.43	104	121	0.41
DSLNT	-	-	-	-	0.17	8	0.25	104	112	0.24
LST c	-	-	0.14	9	0.22	8	0.19	104	121	0.19
3′SL	-	-	0.41	9	0.15	8	0.16	104	121	0.18
LST b	-	-	0.06	9	0.04	8	0.06	101	118	0.06
LST a	-	-	0.01	9	-	-	-	-	9	0.01

Supplementary Table S5: Mean and pooled mean values of individual HMOs for mothers delivering at term belonging to milk group 4 ($Se^{-}Le^{-}$).
Note that values marked with * could not be reproduced by other independent studies and / or were obtained from a few measurements only. Therefore, no pooled mean values were calculated for these HMOs.

	Coppa et al. 2011		Austin et al. 2019		Samuel et al. 2019			
**HMO**	**c_{mean} / g/L**	***n* (samples)**	**c_{mean} / g/L**	***n* (samples)**	**c_{mean} / g/L**	***n* (samples)**	**n_{total} (samples)**	***c* (pooled mean) / g/L**
F-LNH II	0.38	4	0.64	8	0.57	53	65	0.56
LNFP III	-	-	0.87	8	0.51	54	62	0.56
3FL	0.44	4	0.39	8	0.52	54	66	0.50
LNFP V	-	-	0.15	8	0.21	54	62	0.20
LNnDFH	-	-	0.06	14	0.06	54	68	0.06
LNFP VI	-	-	0.04	6	0.03	2	8	0.04
2′FL	-	-	0.02	2	0.02	6	8	0.02
LNFP II	-	-	-	-	0.72*	3	-	-
DFLNH I	-	-	0.05*	1	-	-	-	-
LNDFH I	-	-	-	-	0.03*	5	-	-
LNT	-	-	2.76	8	2.09	54	62	2.17
LNnT	-	-	0.15	8	0.08	48	56	0.09
6’GL	-	-	0.06	8			8	0.06
3‘GL	-	-	0.02	1	0.04	54	55	0.04
DSLNT	-	-	0.45	8	0.43	54	62	0.43
6′SL	-	-	0.44	8	0.38	54	62	0.38
LST c	-	-	0.45	8	0.15	54	62	0.19
3′SL	-	-	0.16	8	0.17	54	62	0.17
LST b	-	-	0.13	8	0.10	52	60	0.11

Preterm-Delivering Mothers

Nakhla et al. [18], Gabrielli et al. [7] and Austin et al. [43] reported individual HMO values of mothers delivering preterm differentiated by milk groups. The studies respectively included 10, 63 and 27 mothers and were performed in the USA, Italy and Switzerland. The respective study durations were 0 – 33 days, 4 – 30 days and 0 – 112 days. Reported mean values of mothers belonging to milk groups 1, 2, 3, and 4 are respectively given in Supplementary Tables S6, S7, S8 and S9. Pooled mean values were calculated as described above.

The pooled mean concentrations of the four most dominant neutral non-fucosylated HMOs are highlighted in blue (if applicable). The pooled mean concentrations of the six most dominant sialylated HMOs are highlighted in violet (if applicable).

Supplementary Table S6: Mean and pooled mean values of individual HMOs for mothers delivering preterm belonging to milk group 1 ($Se^{+}Le^{+}$).
Note that values marked with * could not be reproduced by other independent studies and / or were obtained from a few measurements only. Therefore, no pooled mean values were calculated for these HMOs.

	Nakhla et al. 1999		Gabrielli et al. 2011		Austin et al. 2019			
**HMO**	c_{mean} / g/L	n (samples)	c_{mean} / g/L	n (samples)	c_{mean} / g/L	n (samples)	n_{total} (samples)	c (pooled mean) / g/L
2′FL	1.04	10	5.43	140	1.96	211	361	3.28
LNFP I	0.27	10	1.74	140	0.79	211	361	1.14
LNDFH I	-	-	-	-	1.05	211	211	1.05
3FL	0.71	10	0.62	140	0.61	211	361	0.62
DFL	0.24	10	0.88	140	0.42	211	361	0.59
DF-para-LNnH	-	-	0.50	140	-	-	140	0.50
DF-para-LNH	-	-	0.43	140	-	-	140	0.43
LNFP II	0.21	10	0.40	-	0.39	211	221	0.38
F-LNH II	-	-	0.32	140	-	-	140	0.32
FSLNnH	-	-	0.44	140	0.22	211	351	0.30
LNFP III	0.07	10	0.41	-	0.31	211	221	0.30
DFLNH I	-	-	0.11	140	0.12	211	351	0.11
LNDFH II	0.10	10	0.15		-	-	10	0.10
LNFP V	0.04	10	0.04	140	0.05	211	361	0.05
LNnDFH	-	-	-	-	0.03	211	211	0.03
LNFP VI	-	-	-	-	0.02	211	211	0.02
DFLNH II	-	-	2.71*	140	-	-	-	-
TFLNH	-	-	0.69*	140	-	-	-	-
LNT	0.37	10	5.24	140	0.93	211	361	2.58
LNnT	0.10	10	1.72	140	0.20	211	361	0.79
LNnH	-	-	0.09	140	-	-	140	0.09
LNH	-	-	0.07	140	-	-	140	0.07
A-tetrasacch.	-	-	-	-	0.05	211	211	0.05
6’GL	-	-	-	-	0.03	211	211	0.03
3‘GL	-	-	-	-	0.01	211	211	0.01
DSLNT	-	-	1.10	140	0.31	211	351	0.62
6′SL	-	-	0.72	140	0.31	211	351	0.47
LST c	-	-	0.88	140	0.16	211	351	0.45
LST a	-	-	0.30	140	-	-	140	0.30
3′SL	-	-	0.26	140	0.19	211	351	0.22
LST b	-	-	0.15	140	0.09	211	351	0.11

Supplementary Table S7: Mean and pooled mean values of individual HMOs for mothers delivering preterm belonging to milk group 2 ($Se^{-}Le^{+}$).
Note that values marked with * could not be reproduced by other independent studies and / or were obtained from a few measurements only. Therefore, no pooled mean values were calculated for these HMOs.

	Gabrielli et al. 2011		Austin et al. 2019			
**HMO**	c_{mean} / **g/L**	***n*** **(samples)**	c_{mean} / **g/L**	***n*** **(samples)**	n_{total} **(samples)**	***c*** **(pooled mean) / g/L**
3FL	1.88	72	1.50	57	129	1.71
LNFP II	1.90	72	1.37	57	129	1.67
DF-para-LNH	0.91	72	-	-	72	0.91
DF-para-LNnH	0.55	72	-	-	72	0.55
LNFP III	0.62	72	0.44	57	129	0.54
LNDFH II	0.51	72	-	-	72	0.51
FSLNnH	0.50	72	-	-	72	0.50
F-LNH II	0.36	72	0.36	57	129	0.36
LNFP V	-	-	0.22	57	57	0.22
DFLNH I	-	-	0.03	20	20	0.03
LNnDFH	-	-	0.11	57	57	0.11
LNFP VI	-	-	0.02	40	40	0.02
LNDFH I	-	-	0.01	57	57	0.01
2'FL	-	-	0.01	57	57	0.01
DFLNH II	2.75*	72	-	-	-	-
LNT	2.01	72	1.63	57	129	1.84
LNnT	1.58	72	0.08	57	129	0.92
LNnH	0.08	72	-	-	72	0.08
LNH	0.05	72	-	-	72	0.05
6'GL	-	-	0.03	57	57	0.03
3'GL	-	-	0.01	57	57	0.01
A-tetrasacch.	-	-	0.01	5	5	0.01
DSLNT	1.35	72	0.40	57	129	0.93
LST c	0.73	72	0.09	57	129	0.45
LST a	0.44	72	-	-	72	0.44
6'SL	0.57	72	0.22	57	129	0.41
3'SL	0.26	72	0.18	57	129	0.22
LST b	0.14	72	0.15	57	129	0.14

Supplementary Table S8: Mean and pooled mean values of individual HMOs for mothers delivering preterm belonging to milk group 3 ($Se^{+}Le^{-}$).
Note that values marked with * could not be reproduced by other independent studies and / or were obtained from a few measurements only. Therefore, no pooled mean values were calculated for these HMOs.

	Nakhla et al. 1999		Gabrielli et al. 2011		Austin et al. 2019			
**HMO**	**c_{mean} / g/L**	***n* (samples)**	**c_{mean} / g/L**	***n* (samples)**	**c_{mean} / g/L**	***n* (samples)**	**n_{total} (samples)**	***c* (pooled mean) / g/L**
2′FL	1.18	2	6.43	28	2.64	12	42	5.10
LNFP I	0.34	2	2.62	28	1.43	12	42	2.17
FSLNnH	-	-	0.42	28	-	-	28	0.42
DF-para-LNnH	-	-	0.41	28	-	-	28	0.41
DFLNH I	-	-			0.38	12	12	0.38
F-LNH II	-	-	0.34	28	0.32	12	40	0.33
LNFP III	0.01	2	0.31	28	0.36	12	42	0.31
DFL	0.04	2	0.39	28	0.10	12	42	0.29
3FL	0.011	1	0.22	12	0.22	12	25	0.21
LNFP V	0.01	2	-	-	0.03	4	6	0.02
LNnDFH	-	-	-	-	0.03*	1	-	-
LNDFH II	>0.01*	2	-	-			-	-
LNT	0.12	2	1.66	28	0.80	12	42	1.34
LNnT	0.11	2	1.34	28	0.10	12	42	0.92
LNnH	-	-	0.08	28	-	-	28	0.08
LNH	-	-	0.05	28	-	-	28	0.05
3'GL	-	-	-	-	0.03	12	12	0.03
DSLNT	-	-	1.04	28	0.22	12	40	0.80
LST c	-	-	0.68	28	0.11	12	40	0.51
6′SL	-	-	0.58	28	0.26	12	40	0.48
LST a	-	-	0.31	28			28	0.31
3′SL	-	-	0.22	28	0.16	12	40	0.20
LST b	-	-	0.18	28	0.08	12	40	0.15

Supplementary Table S9: Mean and pooled mean values of individual HMOs for mothers delivering preterm belonging to milk group 4 (Se^-Le^-).

	Gabrielli et al. 2011			
**HMO**	c_{mean} / g/L	n (samples)	n_{total} (samples)	c (pooled mean) / g/L
F-LNH II	0.81	12	12	0.81
FSLNnH	0.57	12	12	0.57
LNFP III	0.55	12	12	0.55
3FL	0.41	12	12	0.41
DFLNnH	0.26	12	12	0.26
LNT	3.18	12	12	3.18
LNnT	1.36	12	12	1.36
LNnH	0.14	12	12	0.14
LNH	0.11	12	12	0.11
DSLNT	1.64	12	12	1.64
LST c	0.60	12	12	0.60
LST a	0.38	12	12	0.38
6′SL	0.34	12	12	0.34
3′SL	0.22	12	12	0.22
LST b	0.16	12	12	0.16
